# Case Report: Rehabilitation of a giant meniscus cyst with a mixed tear

**DOI:** 10.3389/fresc.2024.1483226

**Published:** 2025-01-17

**Authors:** Jing Ji, Yali Wang, Xitao Li, Yuling Wang

**Affiliations:** ^1^ Department of Rehabilitation, Gansu Traditional Chinese Medicine Hospital, Lanzhou, China; ^2^ Department of Rehabilitation, The Sixth Affiliated Hospital of Sun Yat-sen University, Guangzhou, China

**Keywords:** meniscus cyst, meniscus tear, post-arthroscopic rehabilitation, meniscus biomechanics, timing of rehabilitation interventions

## Abstract

Giant meniscus cysts combined with mixed tears are relatively uncommon in clinical practice. The primary objective of rehabilitation is to restore knee joint function and prevent cyst recurrence. In this article, we discuss a series of rehabilitation strategies implemented for a patient who experienced both a giant meniscus cyst and a mixed tear.

## 1 Background

A meniscus cyst is a condition characterized by the accumulation of cystic fluid within the meniscus or beneath the meniscal synovial layer. The cyst is typically located within the joint capsule and its incidence rate is in the range of 1%–8% (1). Although the exact cause is not fully understood, the prevailing view is that synovial fluid in the knee joint accelerates its flow and accumulation due to trauma, subsequently accumulating in the meniscus through a mechanism akin to a check valve, thereby forming a cyst (1, 2). Clinical manifestations of this condition include joint tenderness, locking, and, occasionally, joint fracture (1). Larger meniscus cysts located in the posterior horn may extend into the popliteal fossa, necessitating differentiation from popliteal cysts. The gold standard for distinguishing between these conditions is magnetic resonance imaging (MRI) (3). Currently, the primary treatment methods involve cyst cleaning and meniscus repair through arthroscopy (1).

## 2 Case presentation

**Figure 1:**
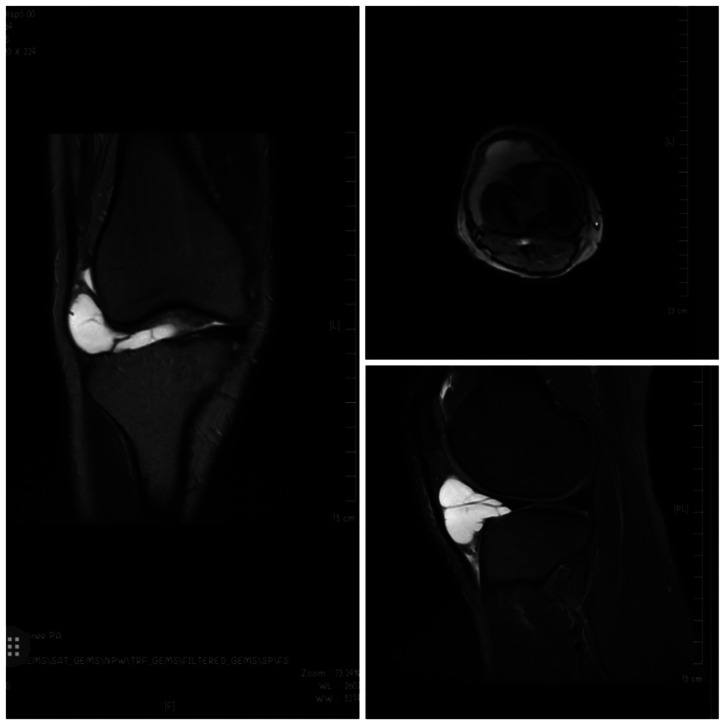
This image represent the MRI findings of the individual.

This case involves a 26-year-old male freelancer who had been experiencing unexplained right knee pain since 2016, with symptoms recurring multiple times. In 2021, an MRI examination revealed a lateral meniscus injury in his right knee, and he underwent conservative treatment at that time. However, in March 2023, the patient reported a significant increase in right knee pain that did not improve with rest. A follow-up MRI examination was conducted, revealing the following findings: (1) injury to the anterior horn of the right lateral meniscus with an associated meniscal cyst formation, and (2) a small amount of effusion in the right knee. A follow-up MRI was conducted as Figure 1.

A physical examination revealed the following findings: tenderness of the medial knee [visual analog scale (VAS) 6/10]; floating patella test (+); McMurray's sign (+); claudication gait [C-Mill setup assessed bilateral lower limb support time: left/right (L/F) 0.679/0.601 s]; Lysholm score 75; a 4 cm × 3 cm cyst was palpated in the right anterolateral knee during knee extension; right knee muscle strength: quadriceps grade 4, hamstring grade 4; and right knee mobility: active/passive range of motion (A/PROM) for flexion 130°/135°, extension A/PROM −5°/0°.

The above images represent the patient’s MRI findings.

### 2.1 Treatment

#### 2.1.1 Surgical treatment

Upon admission, the patient underwent arthroscopic synovectomy of the right knee to address the meniscus and perform a repair using suturing techniques. During the arthroscopy, synovial hyperplasia was observed on both sides of the patella, and a transverse tear was identified in the white zone of the lateral meniscus body. In addition, a cyst had formed on the medial side of the anterior patellar fat pad, extending anteriorly and laterally from the edge of the lateral meniscus, resulting in a large cyst.

As a result, the orthopedists decided to incise the anterior patellar fat pad under arthroscopy and remove approximately 20 ml of viscous yellow cystic fluid. The orthopedists then thoroughly cleaned the anterior horn and body of the meniscus, followed by suturing the lateral meniscus body and anterior horn, as well as the knee transverse ligament and the capsular wall. Some of the proliferative synovial tissue was simultaneously excised, and the lateral meniscus was shaped parallel to its normal anatomy. Finally, the joint cavity was thoroughly rinsed, and 3 ml of sodium hyaluronate were injected into the cavity before suturing the wound and applying a sterile pressure bandage.

#### 2.1.2 Convalescent treatment

In theory, rehabilitation should be implemented immediately postoperatively to avoid cyst recurrence and restore knee joint function.

The main rehabilitation goal for the first week postoperatively was to reduce swelling and control the volume of synovial fluid. Specific measures included the following: (1) raising the lower limbs to a height of approximately 10 cm above the level of the heart and applying an ice pack for approximately 5 min after each knee joint movement to help reduce swelling; (2) applying heatless ultrashort-wave therapy to the affected knee joint to help control the inflammatory response; (3) performing approximately 30 ankle pump exercises every 2 h to promote blood circulation; (4) using a knee brace positioned at 0° to stabilize the joint; (5) physical therapists performed upward, downward, inward, and outward movements on the affected patella to prevent adhesions within the knee joint; (6) stretching of the hamstring muscle and triceps muscles for 30 s per group, repeating three times per group, for a total of three to five times per day, was carried out to prevent knee and ankle joint contractures; and (7) strength training for the quadriceps and hamstring muscles was performed using isometric contractions while supine with a brace to facilitate these isometric activities. Each contraction was held for 10 s and repeated until the individual felt difficulty in controlling the muscles, to avoid excessive exercise that could lead to swelling and metabolic block. This was done three to five times a day.

At 2–4 weeks postoperatively, the patient received rehabilitative training. The training content included the following: (1) the heel was slid while in a supine position (with the foot in a neutral position) to reduce pressure and tension in the meniscus repair area; (2) the active range of motion of the knee joint was gradually increased to 100°; (3) strength training for the gluteus maximus was initiated to help maintain the extended position of the affected knee joint; and (4) an ergometer was used for cyclic training to maintain knee joint mobility and promote synergy between the knee flexion and extension muscles, thereby avoiding cyst recurrence and restoring knee joint function. In theory, immediate postoperative rehabilitation was crucial for optimizing recovery and preventing complications.

### 2.2 Outcome and follow-up

After 16 weeks of rehabilitation and follow-up, the patient's knee joint function recovered well, with no tenderness around the knee joint and no significant abnormalities in movement angle, balance ability, weightbearing status, and gait. The results of various tests 16 weeks postoperatively showed that the patient had achieved the conditions to resume normal life. The specific evaluation results are as follows: the Tampa Scale of Kinesiophobia score was 24 points and the Y balance test results for the left/right relative extension distance was 69%/89% (4). Moreover, the patient's main task was to continue strengthening and improving the muscle strength and knee stability of the right lower limb in the future to ensure safety after returning to normal exercise. Although the patient's functions had not yet been restored to his optimal state, he was very satisfied with his current recovery. The patient started the next stage of training. Two months ago, we communicated online, and he stated that his affected knee was functioning well and had not caused any inconvenience to his daily life (Table 1).

**Table 1:** Follow-up functional assessments.

Follow-up period	A/PROM	Weight bearing	Berg balance scale (BBS)	Gait (C-mill training)	Next stage of action
4ws	95°/102°	Partial	NT	NT	Quadriceps, hamstrings, and gluteal muscle groups (open-chain training) (5, 6)
6ws	114°/125°	Complete	34	L/R step length (%) 48.2%/51.8%, bilateral support phase	Closed-chain activities such as slight squatting against the wall, half lunge, and heel lifting
				L/R 0.628 s/0.601 s	Muscular endurance training
10ws	124°/130°	Complete	47	L/R step length (%) 48.7%/51.3%, bilateral support phase	Using elastic bands, trampolines for balance improvement, transitioning from bilateral to unilateral.
				L/R 0.626 s/0.609 s	Agile running drills (jumping grids), jogging (forward and backward), lateral carioca
16ws	127°/135°	Complete	56	L/R step length (%) 49.3%/50.7%, bilateral support phase L/R 0.633 s/0.631 s	Return to normal life (specific description about patient recovery at next paragraph)

L, left; R, right; 4ws, 4 weeks; 6ws, 6 weeks; 10ws, 10 weeks; 16ws, 16 weeks; NT, not test; A/PROM, active/passive range of motion.

At the beginning of physical therapy, the patient was skeptical about its effectiveness in improving his knee condition, given the long duration of his symptoms. He only began to feel at ease 5 days postoperatively, once the pain and swelling in his knee were well controlled. From that point on, he actively cooperated with our treatment to increase knee joint movement and training to strengthen the muscles. This may delay his knee from regaining full mobility.

## 3 Discussion

Very few people experience large meniscus cysts with mixed meniscus tears as in this case. Repairing the meniscus and postoperative rehabilitation, when combined with surgery, is crucial for restoring normal activity. In this paper, we conducted a 16-week postoperative follow-up on the individual. The summary is as follows.

First, it is important to pay attention to the timing of weightbearing on the meniscus. Patients need to be aware of the size and location of the meniscus tears, as well as the method of repair surgery used. In general, meniscectomy or partial resection can be performed immediately postoperatively. For meniscus repair, patients should maintain non-weightbearing or partial weightbearing for 2–6 weeks postoperatively (7). The physical weight loads on the tibiofemoral joint will exert circumferential pressure on the meniscus and premature loading on the meniscus can affect its healing (8, 9). If the tear occurs in the white zone (the inner third of the meniscus), a weightless state should be maintained for the first 4 weeks. If the tear is stellated, a weightless state must be maintained for the first 6 weeks (9). In this case, we paid particular attention to the tears observed in the lateral meniscus body and anterior horn to ensure that the patient could effectively protect his repaired meniscus after surgery and avoid premature loading.

Second, we should consider the changes in kinetic chain and force loads, as these may impact the force distribution within the knee joint. During the initial rehabilitation training, such as during closed-chain exercises, it is important to keep the patient's knee and ankle joints in a neutral position. Any changes in the angle and rotation of the knee can significantly affect the pressure and tension on the meniscus (10). According to some studies, the maximum pressure on the knee during normal gait is two to three times the body weight, with the meniscus bearing 40%–60% of this load. This pressure increases by 25%–50% if the knee is engaged in angular or rotary activity (11–13). Therefore, we must pay attention to the area of the meniscus tear postoperatively to gradually increase the flexion angle of the knee joint during rehabilitation. If the tear occurs in the red zone (the outer third of the meniscus), the patient can immediately postoperatively bend the knee joint to 90°. However, if the tear occurs in the white zone (the inner third of the meniscus), the range of motion of the knee joint should be moderately limited to below 70° during the initial postoperative period (the first 2 weeks) (14, 15).

Third, in the process of enhancing muscle strength, attention should be paid to avoiding excessive strengthening of the quadriceps femoris muscle. The quadriceps muscle is connected to the meniscus through pathological ligaments and actively extending the knee may pull the meniscus forward (15, 16). Simultaneously, the femoral condyle exerts anteroposterior pressure on the meniscus (10). Therefore, in the early stages of rehabilitation, the patient's active knee extension angle should not be excessive, and the focus should be on strengthening the myodynamia of popliteal muscle. This approach helps promote scar formation and regeneration of the meniscus under appropriate pressure and tension.

In conclusion, during the rehabilitation process after meniscus repair, it is crucial to carefully control the load applied to the meniscus to ensure the integrity of the repaired meniscus and achieve optimal rehabilitation results. In this case, the tear was located at the anterior horn and body of the meniscus; therefore, lower limb weightbearing should be limited within 5 weeks postoperatively to prevent overloading the affected meniscus and active knee flexion should be postponed. For patients with meniscus cysts, various measures should be taken to control the volume of synovial fluid to prevent recurrence of the cyst.

Although this article summarizes the rehabilitation process of a single case and discusses the photomechanical analysis of the impact of knee flexion and extension on the meniscus, it does not provide quantitative methods for diverse patients or propose a universal therapeutic method. This limitation may reduce its applicability as a reference for peers encountering similar cases. Therefore, readers are recommended to focus on the discussion section, which offers insights into the biomechanical properties of the meniscus and knee joint, allowing for a deeper understanding while saving time.

## Data Availability

Publicly available datasets were analyzed in this study. This data can be found here: nothing.
